# Electrostatic recognition in substrate binding to serine proteases

**DOI:** 10.1002/jmr.2727

**Published:** 2018-05-22

**Authors:** Birgit J. Waldner, Johannes Kraml, Ursula Kahler, Alexander Spinn, Michael Schauperl, Maren Podewitz, Julian E. Fuchs, Gabriele Cruciani, Klaus R. Liedl

**Affiliations:** ^1^ Institute of General, Inorganic and Theoretical Chemistry, and Center for Molecular Biosciences Innsbruck (CMBI) University of Innsbruck Innsbruck Austria; ^2^ Laboratory of Chemometrics, Department of Chemistry University of Perugia Perugia Italy

**Keywords:** electrostatic similarity, encounter complex, molecular interaction fields, protease, substrate, substrate recognition

## Abstract

Serine proteases of the Chymotrypsin family are structurally very similar but have very different substrate preferences. This study investigates a set of 9 different proteases of this family comprising proteases that prefer substrates containing positively charged amino acids, negatively charged amino acids, and uncharged amino acids with varying degree of specificity. Here, we show that differences in electrostatic substrate preferences can be predicted reliably by electrostatic molecular interaction fields employing customized GRID probes. Thus, we are able to directly link protease structures to their electrostatic substrate preferences. Additionally, we present a new metric that measures similarities in substrate preferences focusing only on electrostatics. It efficiently compares these electrostatic substrate preferences between different proteases. This new metric can be interpreted as the electrostatic part of our previously developed substrate similarity metric. Consequently, we suggest, that substrate recognition in terms of electrostatics and shape complementarity are rather orthogonal aspects of substrate recognition. This is in line with a 2‐step mechanism of protein‐protein recognition suggested in the literature.

## 1 INTRODUCTION

In humans, more than 560 different genes code for proteases^1^ constitute nearly 3% of all approximately 19 000 human genes^2^; therefore, proteases have been investigated as drug targets extensively.^3^ Many protease inhibitors are already very successful drugs, eg, to control blood coagulation,^4^ to treat hypertension and diabetes,^5^ to fight cancer, and to combat viral diseases like HIV^6^ and Hepatitis C.^7^ However, protease promiscuity and overlapping specificity profiles are major problems that have to be overcome in most protease inhibitor drug design efforts.^8^, ^9^

Proteases catalyze the hydrolysis of peptide bonds through acceleration of the nucleophilic attack on the peptide amide group, which would otherwise be kinetically stable.^10^ To fulfill their biological function, proteases have to bind their native substrates and stabilize the transition state for hydrolysis of the peptide bond.^11^ Promiscuity and specificity of this recognition process not only exhibit striking differences between different proteases, but also show large variations within the binding cleft of an individual protease.^12^, ^13^, ^14^ Obviously, evolution had to tackle quite different tasks—on the one hand, designing proteases that are able to digest more or less every peptide that they encounter, and on the other hand, designing proteases within a signaling cascade, that should specifically recognize the subsequent member of the signaling chain to ensure the proper transmission of the signal.^15^ This evolutionary pressure led to proteases ranging from highly promiscuous to extremely specific. Surprisingly, these extremes can occur within the same family of evolutionarily related proteases, eg, the Chymotrypsin family of serine proteases.^16^

**Figure 1:**
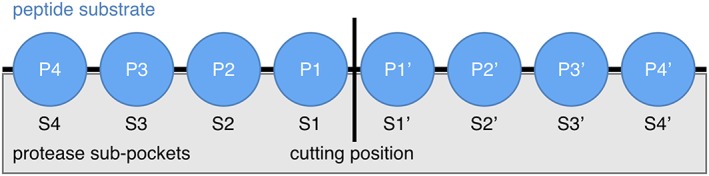
Peptide substrate amino acid (Pi and Pi′) and protease subpocket enumeration (Si and Si′) with respect to the cutting position (vertical line). The N‐terminal side of the substrate is located on the left

The promiscuity and specificity of substrate recognition are very often not spread evenly along the binding cleft. Proteases often prefer certain substrate amino acids in given distances to their catalytic center. Half a century ago, Schechter and Berger^17^ suggested a convention to denote the peptide substrate amino acids (P4 to P4′) and the subpockets (S4 to S4′) within the binding cleft around the scissile bond (cf. Figure 1).

Several methods have been published to describe and localize promiscuity and specificity of the protease binding interface, thus facilitating a comparison of individual proteases.^18^, ^19^, ^20^, ^21^ Our cleavage entropy metric^14^, ^22^ is based on substrate data deposited in the MEROPS database^23^ and quantifies the specificity of peptide recognition in each subpocket. To compare proteases based on their substrate recognition, we developed a metric that considers the positional abundance of individual amino acids.^24^ These methods allow the investigation of localized regions of promiscuity and specificity in the binding interface of proteases and the analysis of their thermodynamic properties. Thus, different subpockets of the same protease as well as binding clefts of different proteases can be studied and compared.

**Figure 2:**
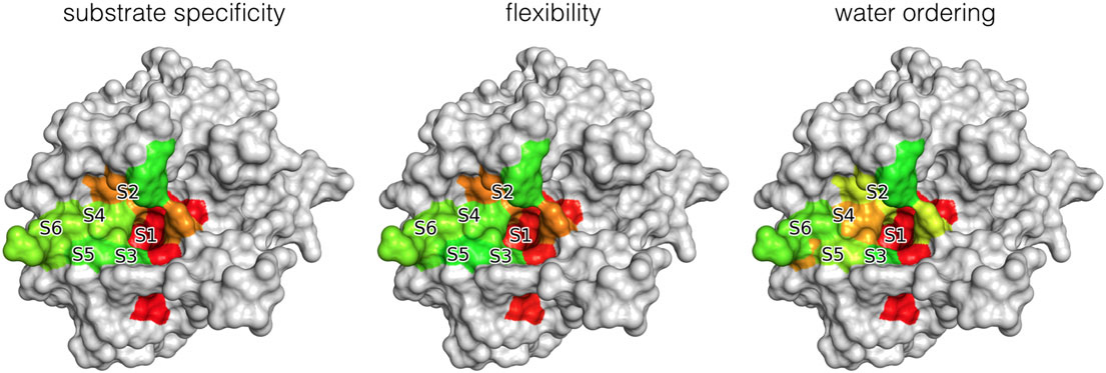
Correlation of substrate specificity with backbone flexibility and orientational ordering of water molecules in the non‐prime site (S6‐S1) of Thrombin's binding cleft (ranging from red—specific, rigid and ordered, via yellow to green—promiscuous, flexible and disordered) 27

Research on snake venom metalloproteases revealed strong hints that their promiscuity is linked to their flexibility.^25^ Likewise, we found that Caspases^26^ and Thrombin^27^ display strong correlations between flexibility and promiscuity. For Thrombin, this correlation translates into ordering processes of water molecules in the binding interface. Regions of specificity show ordered water molecules in the interface, whereas regions of promiscuity tend to have more disordered water molecules in the first solvation sphere (Figure 2). On the other hand, enthalpic contributions to hydration of the S1 and S4 to S6 are almost identical. Thus, dynamic, and therefore entropy of hydration, contributes strongly to the recognition of substrates in Thrombin.

Electrostatic interactions are quite different from other contributions of substrate recognition as they are long‐range interactions that change little with small differences in distance.^28^, ^29^ This has several important consequences. Obviously, a more continuous distance dependence varies less with conformational changes, much in contrast to shape‐dependent recognition like van der Waals interactions and recognition that relies on precise exit vectors like hydrogen bonds. On the contrary, due to the long‐range character of electrostatic interactions, assigning them to specific subpockets is more challenging.

Calculating differences in electrostatic molecular interaction fields (eMIFs) of proteins is a rather challenging task. Many different approaches exist, and all of them have a significant impact on the result. Differences in the handling of the solvent and the solute, either implicit as a continuum or explicit, can yield highly different results.^30^ When using an implicit model, it is also not trivial to assign each point on the grid a certain value for the dielectric constant. This problem is irrelevant for high distances to the solute but can yield errors for points close to it.^31^ Furthermore, differences in handling multipoles will also introduce differences in the results.^32^ The biggest error, however, is included when using different protonation states for the model, as introducing an extra charge, or removing one, changes the entire electrostatic field significantly.

In a previous study, we used GRID‐probes that test van der Waals interactions and electrostatics simultaneously. Even taking into account conformations extracted from molecular dynamics trajectories, we could only achieve limited correlation with substrate recognition.^33^

To predict the specificity of proteases, Pethe et al^34^ used a structure‐based approach that ranks possible substrates according to interaction energies and reorganization penalties. Their scheme outperforms conventional methods that focus solely on knowledge‐based prediction of substrate preferences.

Okun and Chen compared proteases with a statistical model. They calculated electrostatic similarities using a volumetric overlay of isopotentials.^35^

In PIPSA,^36^ the Hodgkin index is used to compare different Molecular Interaction Fields of proteins. The program was also used by Henrich et al^37^ to compare the electrostatic similarity of the 3 proteases, Trypsin, Thrombin, and uPA.

Various approaches are already available that compare binding sites,^38^ often for the purpose of off‐target prediction and drug repurposing.^39^ Such methods rely on molecular interaction fields (MIFs), eg, BioGPS^40^, ^41^ and IsoMIF,^42^ on shape and physicochemical properties of the surface, eg, protein functional surfaces,^43^ on graphs representing the 3D atomic similarities, eg, IsoCleft^44^ or on fingerprints describing the binding sites, eg, PocketMatch.^45^ In several data bases, properties of binding sites are stored for comparison, such as pseudocenters with projected physicochemical properties in CavBase,^46^, ^47^, ^48^ in CavSimBase^49^ and in SiteEngine,^50^ sequence and structural similarity in CPASS,^51^ position of functional groups in SuMo,^52^ or surface geometrics and electrostatics in eF‐site.^53^

**Figure 3:**
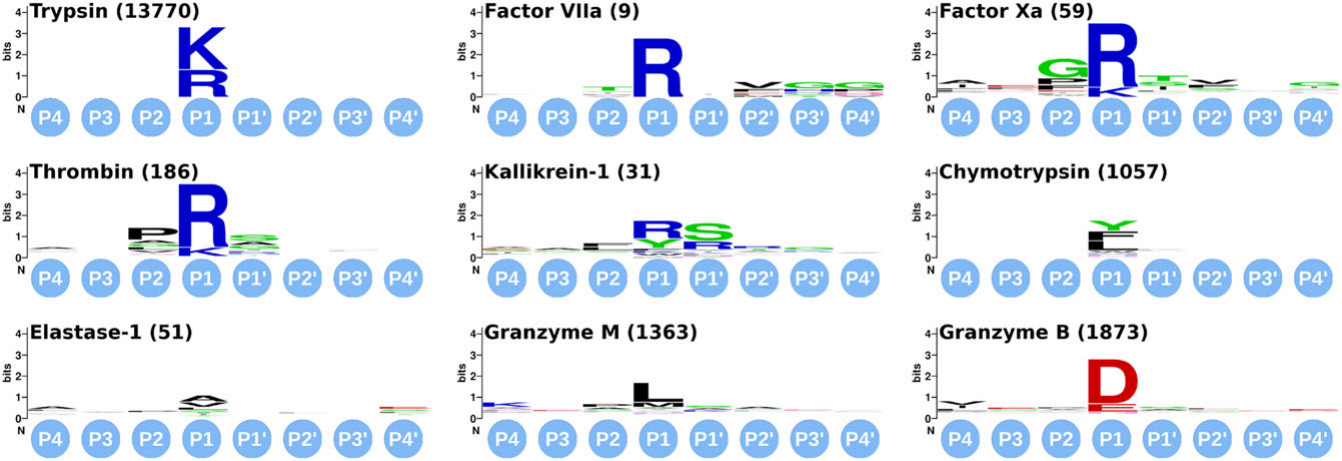
Cleavage site sequence logos of substrate data used for generating substrate preference similarity metric. Numbers in brackets indicate the number of substrates filed in the MEROPS database for each protease. The logos were generated with WebLogo 54 (the underlying data is supplied in the Supporting Information)

However, most of these methods are not meant to compare structurally very similar cavities as found in our set of chymotrypsin‐like proteases (Figure 3), or define the binding site without ligand information. Therefore, we chose to implement our own method optimized to compare similar binding interfaces and able to compare these interfaces based on a distance criterion to a ligand as described in the methods section below in detail.

## 2 METHODS

### 2.1 Electrostatic substrate preferences

We extracted and isolated the electrostatic contributions and the shape recognition contributions in the substrate preference similarity metric that we defined previously.^24^ We achieved this goal by binning the amino acid residues according to their electrostatic properties into positively charged (K, R, H), negatively charged (D, E), and neutral amino acids (G, P, A, V, L, I, M, F, Y, W, S, T, C, N, Q). In this way, we split off shape‐dependent and size‐dependent aspects of substrate recognition and focus solely on electrostatic recognition and specificity. The shape‐dependent aspects of substrate recognition can be studied individually for each of the 3 bins, especially within the bin of neutral amino acids. However, this aspect is beyond the scope of the current study. Considering histidine as a positively charged amino acid is somewhat arbitrary, but in line with the usual classification in sequence logos.^54^ Nevertheless, histidine is rather underrepresented in substrate data (1.7%); thus, this choice does not influence the analysis significantly (correlation data is shown in the Supporting Information).

For each of the 9 proteases under investigation, we extracted substrate data from the MEROPS database^23^ (Trypsin S01.151, Factor VIIa S01.215, Factor Xa S01.216, Thrombin S01.217, Kallikrein‐1 S01.160, Chymotrypsin S01.001, Elastase‐1 S01.153, Granzyme M S01.139, Granzyme B S01.010) for substrate residues P4 to P4′.

For each substrate position, amino acids are assigned to 1 of the 3 bins according to their electrostatic properties. For each substrate position, a vector is constructed via Equation (1).

(1) $$v_{\mathrm{Pi}}=\left(N_{\text{positive}}^{\mathrm{Pi}}=\frac{\sum_{a=1}^{3}n_{a}^{\mathrm{Pi}}}{\sum_{a=1}^{3}p_{a}},\;N_{\text{negative}}^{\mathrm{Pi}}=\frac{\sum_{a=1}^{2}n_{a}^{\mathrm{Pi}}}{\sum_{a=1}^{2}p_{a}},\;N_{\text{neutral}}^{\mathrm{Pi}}=\frac{\sum_{a=1}^{15}n_{a}^{\mathrm{Pi}}}{\sum_{a=1}^{15}p_{a}}\right)$$

In Equation (1), *N*_*bin*_^*Pi*^ is the score in 1 of the 3 electrostatic bins (positive, negative, or neutral) of substrate position *Pi*, *a* the index of the amino acids in 3 different bins, *n*_*a*_^*Pi*^ the number of occurrences of amino acid a in the substrate position *Pi*, and *p*_*a*_ the natural occurrence of amino acid a. This weighting with the natural occurrence of the amino acids in human proteins^55^ results in an intrinsic normalization of the bins.

Concatenation of vectors **v**_Pi_ for substrate positions P4 to P4′ and bin is summarized into a vector of dimension 3·8 = 24. This vector is subsequently normalized to 1 and contains the electrostatic substrate preferences for each protease.

(2) $$v_{\text{Protease}}=\frac{\left(v_{P4},\;\ldots ,\;\;v_{P4\prime }\right)}{\left\|\left(v_{P4},\;\ldots ,\;\;v_{P4\prime }\right)\right\|}$$

The electrostatic substrate similarity of 2 proteases is calculated by forming the scalar product between the respective electrostatic substrate preference vectors. A scalar product of 0 indicates orthogonal substrate preferences. A scalar product of 1 implies identical electrostatic substrate preferences, as found when comparing an individual protease with itself, ie, the electrostatic substrate preference. Additionally, the product of every single line of the vector can be interpreted as contribution corresponding to either positive, negative, or neutral substrate residues at every single substrate position. The sum of individual substrate positions indicates the overall contribution of positive, negative, and neutral substrate residues.

### 2.2 Electrostatic molecular interaction fields (eMIFs)

X‐ray structures for all 9 proteases were downloaded from the PDB (Trypsin 1PQ7,^56^ Factor VIIa 1KLI,^57^ Factor Xa 1C5M,^58^ Thrombin 4AYY,^59^ Kallikrein‐1 1SPJ,^60^ Chymotrypsin 4CHA,^61^ Elastase‐1 1QNJ,^62^ Granzyme M 2ZGH,^63^ Granzyme B 1FQ3^64^). For Thrombin and for Granzyme M, structures with a ligand were chosen to ensure a conformation of the active form.^65^, ^66^ For structures containing a ligand, the ligand was removed.

The structures were aligned with respect to their Cα atoms, and all structures were protonated with Protonate3D^67^ and prepared with MOE.^68^ The catalytic histidine (His‐57) was chosen to be uncharged in all structures.

Using the program GRID,^69^, ^70^ 3 molecular interaction fields (MIFs) were calculated on a grid for the entire binding interface of the proteases. For each probe, a grid spacing of 1 Å was used. For the first MIF, a hydrophobic H‐probe was used in order to characterize the shape of the binding cleft. It was further restricted by a distance criterion (5 Å) to ligands (P4 to P4′) in aligned peptide complex structures, ie, 1DE7, 3LU9 (Thrombin), and 2ZGH (Granzyme M). Only grid points that fulfill the distance criterion and show favorable interactions (<0 kcal/mol) with the H‐probe were used in the further calculations of the eMIFs. In order to minimize van der Waals interactions and focus on electrostatic contributions alone, the eMIFs were calculated with user‐defined GRID probes with charges of +1 and −1. Both, the van der Waals radius of the probes and the cutoff for the van der Waals interactions, were set to their smallest allowed input values of 0.01 and 3 Å, respectively, basically switching of the van der Waals interactions. Only points of the eMIFs that show favorable interactions (<0 kcal/mol) were kept for further calculations. Obviously, the points of favorable interactions for the positive probe are points of unfavorable interactions for the negative probe and vice versa. Thus, the final eMIFs are represented by a grid, of which the points need to have a negative energy (favorable interaction) and furthermore have to be a subset of the previously selected grid points of the H‐probe (proximity to the ligand, no overlap with the protease itself).

The proteases and their eMIFs were realigned slightly by overlaying the weighted center of grid points of the H‐probe MIFs and aligning the first eigenvector^71^ of the H‐probe MIFs tensor of inertia, ie, the one corresponding to the largest eigenvalue. Due to the high similarity of the binding clefts, this procedure resulted in an excellent alignment. Thus, the second and third eigenvalues were not used for further refinement, avoiding problems due to their near degeneration. The resulting alignment was consequently used for analyses with the electrostatic GRID probes.

The overlap of the eMIFs corresponding to the same charge was calculated using spherical Gaussian functions with a σ of 2 Å centered at the grid points, according to Equation (3). We tested several options for the width of the Gaussian function, ranging from 1 to 2 Å but found surprisingly little impact on the resulting correlation with experimental substrate data, which is in line with the long‐range nature of electrostatic interactions, the results of these calculations are summarized in the supporting info.

(3) $$O=N_{A}N_{B}\cdot {\left(\frac{\pi \sigma_{A}^{2}\sigma_{B}^{2}}{2\left(\sigma_{A}^{2}+\sigma_{B}^{2}\right)}\right)}^{\frac{3}{2}}\cdot \exp\left(-\frac{{\left(r_{A}-r_{B}\right)}^{2}}{2\left(\sigma_{A}^{2}+\sigma_{B}^{2}\right)}\right)$$

In Equation (3), N_A,B_ is the height of the spherical Gaussian function, σ^2^_A,B_ is the variance of the spherical Gaussian function, and **r**_A,B_ is the center of the spherical Gaussian function, for A and B, respectively.

## 3 RESULTS

### 3.1 Electrostatic substrate similarities

Electrostatic substrate similarities show whether 2 proteases cleave similar substrates or whether they have opposing substrate preferences. The substrates are only characterized by their charge—positive, neutral, or negative. The shape of the amino acids is neglected, so, eg, the recognition of glutamate and aspartate is considered to be exactly equivalent. The electrostatic substrate similarities can be broken down to the contributions of the single substrate positions that sum up to the total value. The contributions to self‐similarities reflect the electrostatic substrate preference of single proteases and are normalized to give a total of 1.

**Figure 4:**
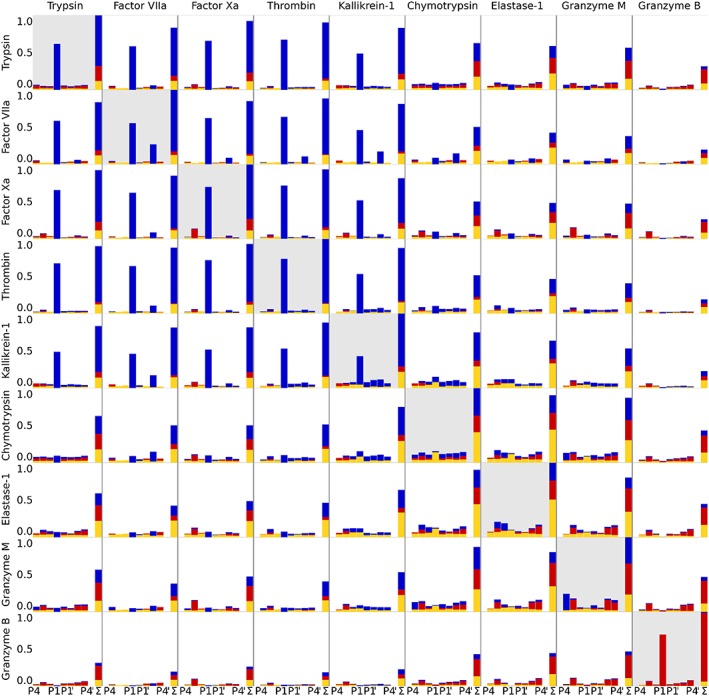
The heights of the bars indicate the electrostatic substrate similarities between all 9 investigated proteases, ranging from P4 to P4′, and the resulting sum (Σ) on the right. Blue represents favoring of positively charged amino acids, yellow neutral ones, and red negatively charged ones. The self‐similarities are depicted as diagonal entries and placed on a gray background in the symmetric matrix

Figure 4 gives an overview of the similarities between 2 proteases in the substrate space after binning the amino acids, as described earlier. Our metric detects prominent similarities among the proteases with high preference for positively charged residues in the S1 subpocket, ie, Trypsin, Factor VIIa, Factor Xa, Thrombin, and Kallikrein‐1. The same holds true for the proteases reading primarily neutral amino acids, ie, Chymotrypsin, Elastase 1, and Granzyme M. Granzyme B, the only protease of our set that predominantly reads negatively charged amino acids, is singled out by our metric and shows low similarities to all other proteases.

Electrostatic substrate preferences are not only highlighted for the S1 subpocket, but also for all other subpockets. For example, the electrostatic substrate preferences of Granzyme M reveal a propensity for negatively charged residues in the subpockets S3, S3′, and S4′ that is hardly identifiable in the cleavage site sequence logos (Figure 3). Among the proteases that prefer positively charged amino acids in S1, Factor VIIa and Kallikrein‐1 are quite different in the substrate preferences in that subpocket, yet they share a preference for positively charged substrate amino acids in S3′. Granzyme B, favoring negatively charged substrate residues over large parts of its binding site, shows generally only minimal similarity with the proteases that prefer positive residues in the S1 subpocket. Still within these, the largest electrostatic substrate similarity with Granzyme B is determined for Trypsin. While Trypsin is very specific for positive amino acids in S1, in remote subpockets, it accepts negatively charged residues. This peculiarity is highlighted when compared with Granzyme B.

### 3.2 Electrostatic molecular interaction fields (eMIFs)

**Figure 5:**
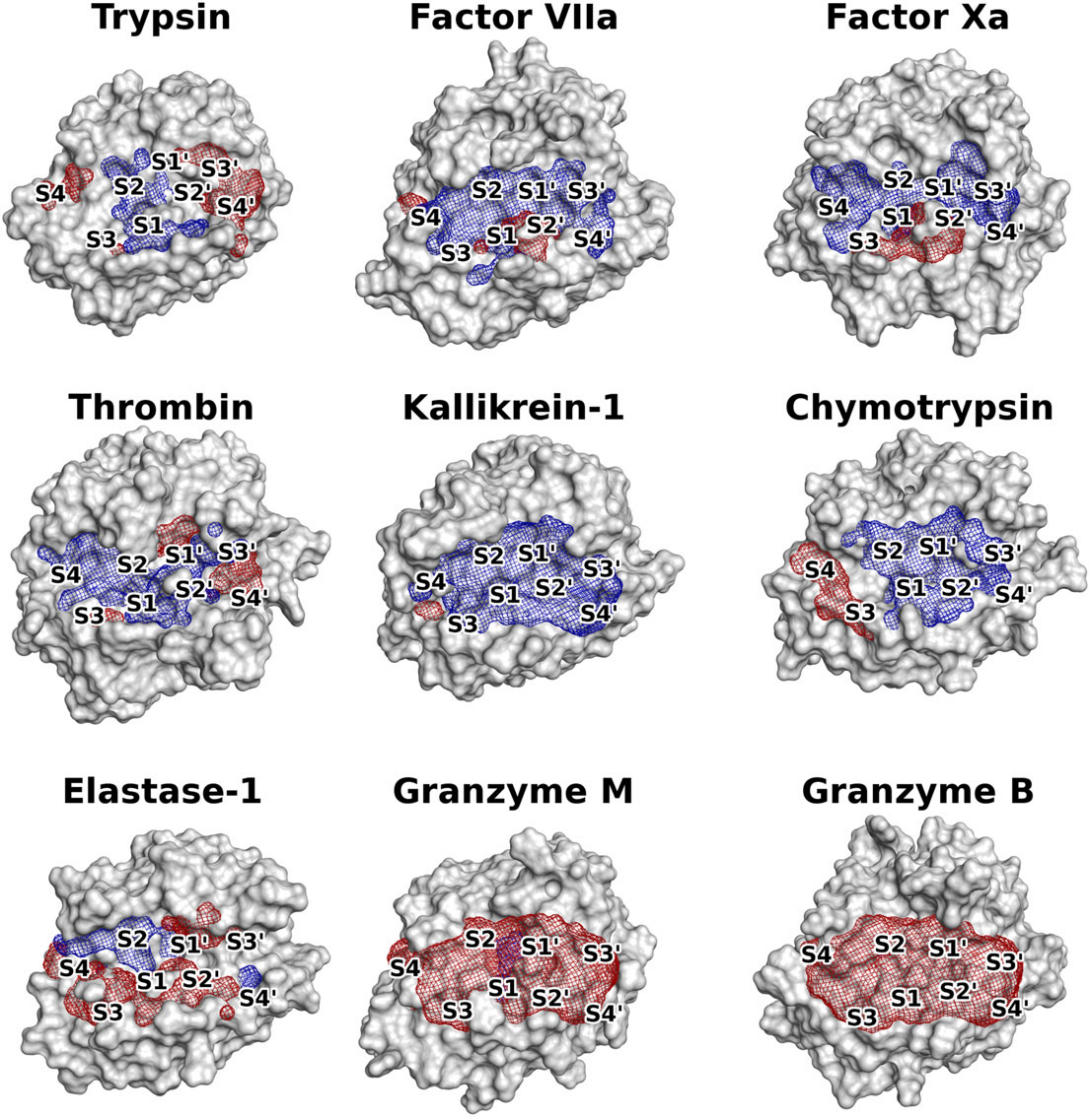
The electrostatic molecular interaction fields (eMIFs) are shown for the 9 investigated proteases. The interactions with the positive probe are depicted in blue, whereas the interactions with the negative probe are depicted in red. A cutoff of −3 kcal/mol was used for the visualization of the fields

With the negatively and the positively charged GRID probes, the eMIFs of the proteases can be determined (Figure 5).

Trypsin shows favorable interactions with the positive probe in its S1 subpocket, while in the more peripheral S4 and S4′ subpockets, it prefers the negative probe. Factor VIIa, Factor Xa, Thrombin, and Kallikrein‐1 favor the positive probe in large parts of their binding clefts. Chymotrypsin has a dyadic eMIF as in the prime site it interacts favorably with the positive probe and towards the outer non‐prime site with the negative probe. In the S2 and the S4′ subpocket, Elastase‐1 favors the positive probe, while in the rest of the prime site and in S3 it favors the negative probe. Both Granzymes seem to show a completely negative eMIF, but on closer inspection Granzyme M favors the positive probe in S1′, the corresponding eMIF is hidden below a layer of grid points favoring interactions with the negative probe.

### 3.3 Electrostatic substrate preferences and electrostatic molecular interaction fields

A joint view of electrostatic substrate preferences and eMIFs of a protease demonstrates the presence of similar patterns in both metrics. In subpockets that are associated with a substrate readout preference for positively charged residues, strong interactions with the positive probe are present, while in subpockets with substrate readout preference for negatively charged amino acid residues the negative interactions predominate. Commonly, where no strong electrostatic substrate preferences are detected, no strong electrostatic interactions can be found.

**Figure 6:**
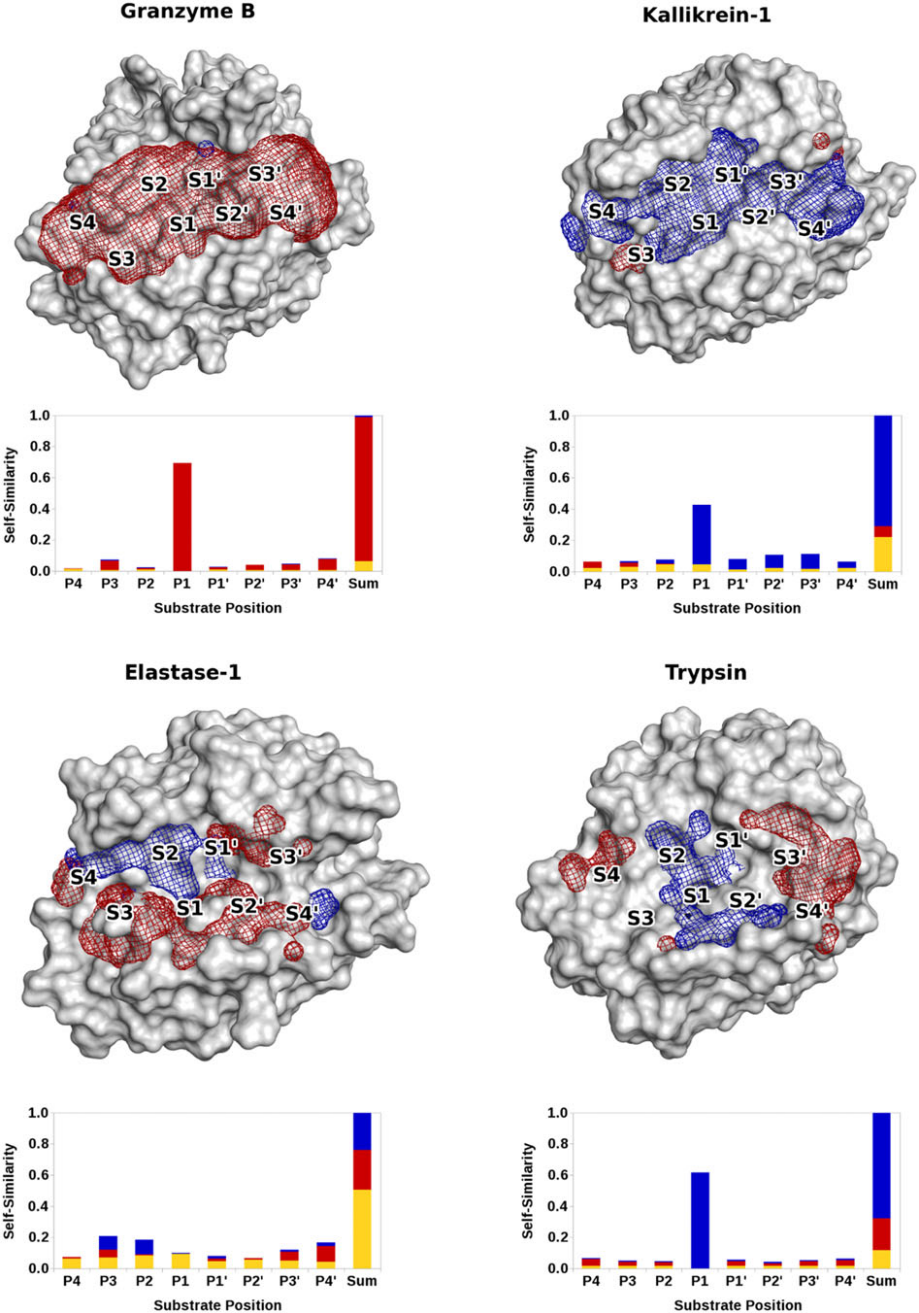
The eMIFs of Granzyme B, Kallikrein‐1 (top), Elastase‐1 and trypsin (bottom) for the positive (blue) and the negative probe (red), using a cutoff of −3 kcal/mol and the electrostatic substrate preference for positive amino acids (blue), neutral amino acids (yellow), and negative amino acids (red). Substrate self‐similarities are in general well reflected by the eMIFs

The eMIFs of Granzyme B show the same pattern as the electrostatic substrate preference (Figure 6), ie, favorable interactions with the negative probe are visible over the entire binding site. In the electrostatic substrate preference, the same trend can be observed.

For Kallikrein‐1, primarily favorable interactions with the positive probe are visible. Substantial areas where the negative probe is favored can be found only in the outer regions of the non‐prime site, ie, the S3 subpocket. Again, this is very well reflected by the electrostatic substrate preference, as in the S3 and S4 subpockets a propensity for negatively charged amino acids can be seen.

For Elastase‐1, the eMIFs reflect the electrostatic substrate preference very well. As the S1 subpocket is specific for neutral amino acids, practically no electrostatic interactions are visible. The prime site substrate self‐similarities show preferences for the negative probe, whereas the non‐prime site varies more in electrostatic preferences. The eMIFs correspond very well with this. In the S3 subpocket, which is to be rather unspecific in terms of electrostatics, both interactions can be observed, although the positive eMIF at that position is barely visible because it is hidden behind the negative one.

In the S1 subpocket of Trypsin, positively charged amino acids are strongly favored, which is also visible in the eMIF. On the periphery of the binding site, the protease starts favoring negatively charged amino acids, which is also mirrored very well by the calculated eMIFs, where the negative eMIF starts to dominate around the S3′ and S4′ subpockets.

### 3.4 Electrostatic substrate similarities and eMIF overlaps

**Figure 7:**
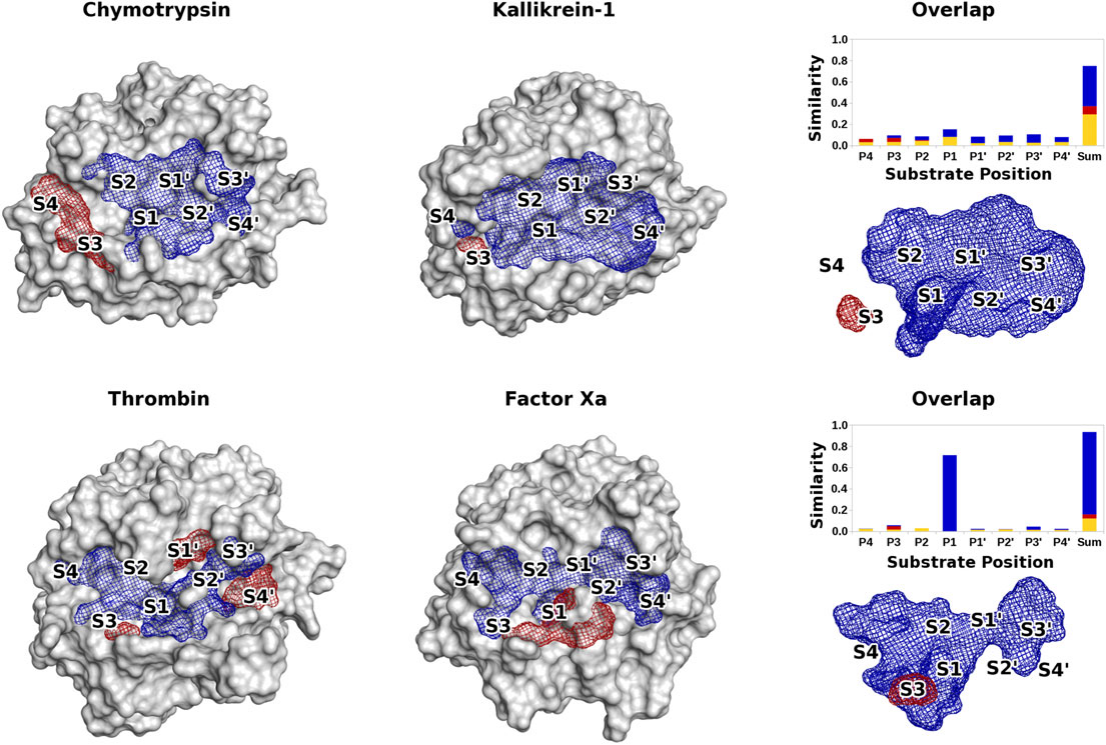
eMIFs, eMIF overlap and electrostatic substrate similarity for chymotrypsin and Kallikrein‐1 (top) and thrombin and factor Xa (bottom): The eMIFs and their overlaps are depicted in blue (positive probe) and red (negative probe). The eMIF overlap was calculated at a cutoff of 0 kcal/mol and visualized at a cutoff of 50 (kcal/mol) ^2^ . The eMIF overlap on the right is depicted without protein surfaces, revealing overlapping eMIFs deep in the S1 subpocket hidden by the protein surfaces on the left. Above the overlap eMIF, the substrate similarity for the proteases is depicted for the positively charged amino acids (blue), for the neutral ones (yellow) and for the negatively charged ones (red)

Calculation of the overlap between the eMIFs of different proteases establishes areas where the same interactions prevail and facilitates the comparison with the electrostatic substrate similarities. Some representative examples of this comparison between eMIF overlap and electrostatic substrate similarities are shown in Figure 7.

Considering Chymotrypsin and Kallikrein‐1 (Figure 7), the differences in substrate similarity can easily be explained by the eMIFs. Both proteases favor positively charged and neutral amino acids in nearly the entire binding cleft. However, on the non‐prime site, Kallikrein‐1 favors negative amino acids in the S3 subpocket. This hotspot is also visible in the overlap eMIF of the 2 proteases. On the entire prime site, the eMIF overlap shows favorable positive interactions, as does the substrate similarity.

Thrombin and Factor Xa are the most similar ones in their electrostatic substrate readout among our set of proteases. Their eMIFs vary only very little (Figure 7). There are small differences in the subpockets on the outer regions of the prime site as well as farther away on the non‐prime site. But near the S1 subpocket, the differences of the eMIFs are negligible.

**Figure 8:**
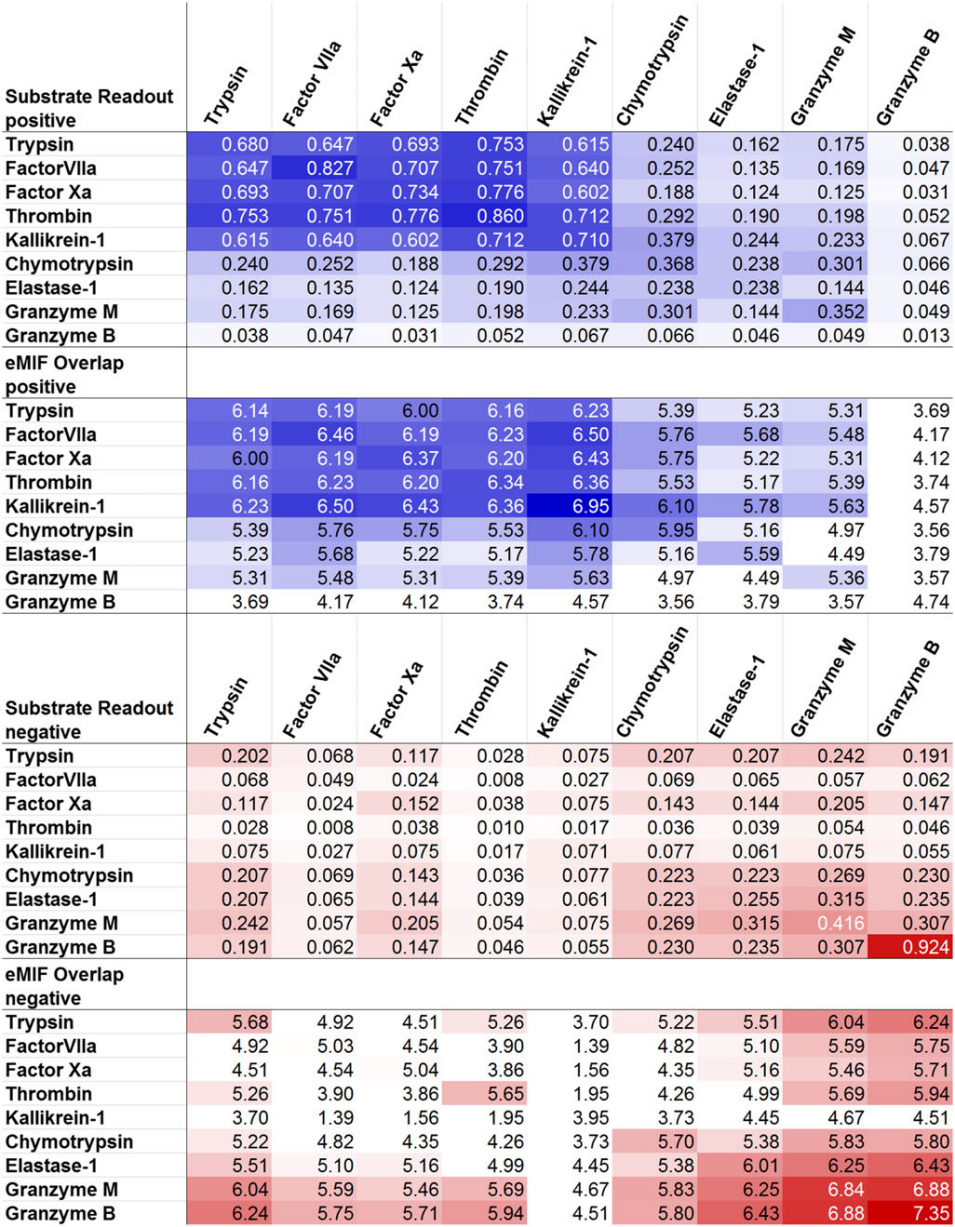
Substrate similarity and eMIF overlap for positive (top) and negative (bottom) substrate space and probe are compared. High similarity and overlap are shaded in dark blue (positive) and dark red (negative), respectively, while low similarity and overlap are depicted on a white background (both). For readability purposes, the eMIF overlap was scaled logarithmically

An overview of the similarities of the 9 proteases in positive substrate readout and positive eMIF overlap is given in the upper part of Figure 8. The lower part of Figure 8 is the corresponding equivalent showing the negative part of the substrate similarities and the eMIF overlaps from the negative GRID probe. The matrices of the substrate similarities match the electrostatic parts of the total substrate similarities already shown in Figure 4.

At first glance, proteases that are similar in electrostatic substrate readout are also similar in their eMIF similarity. To quantify the correlation of the similarity in substrate space and the eMIF similarity, a Mantel test was performed,^72^, ^73^ resulting in a Pearson correlation of 0.82 for the positive and 0.57 for the negative probe, respectively. The correlation for the positive probe is excellent. The correlation for the negative probe still is surprisingly high, as the binding clefts of most proteases of our set are dominated by interactions with the positive probe with minor contribution of the negative probe.

Furthermore, the similarity of the eMIFs at the binding site of the different proteases was also calculated using the webPIPSA server^36^, ^74^ and the APBS method^75^ for calculation of the eMIF (Figure S8 in the Supporting Information). In webPIPSA, we defined the selection of the binding site via the coordinates centered between the Cαs of the W215, the catalytic serine (S195) and the E192, as well as a radius of 17 Å. In general, both approaches are in good agreement, with a few differences between them. The most striking discrepancy is the apparent dissimilarity of Factor VIIa in the webPIPSA results to most of the other proteases, primarily reading positively charged amino acids in the S1. While the presented approach finds that the eMIF of Factor VIIa is rather similar to all these proteases. This difference could be due to the different definition of the binding interface, or to the different method to calculate the similarity.

## 4 DISCUSSION

In a previous study, a conclusive correlation between the knowledge‐based protease substrate specificity and physics‐based enthalpic aspects of the binding cleft of proteases^33^ was hampered by simultaneously considering van der Waals interactions and electrostatics. With the improved approach presented in this work, where we separate electrostatics from van der Waals interactions, in contrast a high correlation is found for electrostatic substrate preferences and eMIFs. This shows that electrostatic recognition is a major factor in protease substrate recognition in all proteases of the Chymotrypsin family investigated here. This is in line with previously published work focusing on Thrombin by Huntington^76^ and on Chymotrypsin C by Batra et al.^77^ The most important aspect of our breakthrough here seems to root back to focusing only at electrostatics, both in characterizing protease readout and interaction profiles. Mechanisms of electrostatic substrate recognition seem to be inherently different from other mechanisms of substrate recognition. Due to their strong nature and their long‐range behavior, electrostatic interactions behave quite differently from other aspects of substrate recognition, which are dominated by shape complementarity.^78^, ^79^ Electrostatic interactions are not only strong and long ranging, but compared with van der Waals interactions vary relatively little with small changes in distance. Hence, it is not surprising that considering only static X‐ray structures already yields a very high correlation between electrostatic substrate similarities and electrostatic interaction field similarities. Flexibility of the binding interface is influencing electrostatics only to a minor extent. Electrostatic contributions would vary substantially solely with major conformational changes and, obviously, with differences in protonation or ion coordination. The long‐range behavior and continuous nature of electrostatic interactions also impede allocating electrostatic contributions to subpockets of the binding clefts. By correlating the electrostatic substrate recognition with the electrostatic interaction field of the entire binding cleft, we avoid the non‐trivial task of apportioning the binding cleft into subpockets. Still, for an efficient substrate prediction, which however is beyond the scope of this study, such a partitioning of electrostatic contributions to subpockets of the binding cleft would be highly desirable.

**Figure 9:**
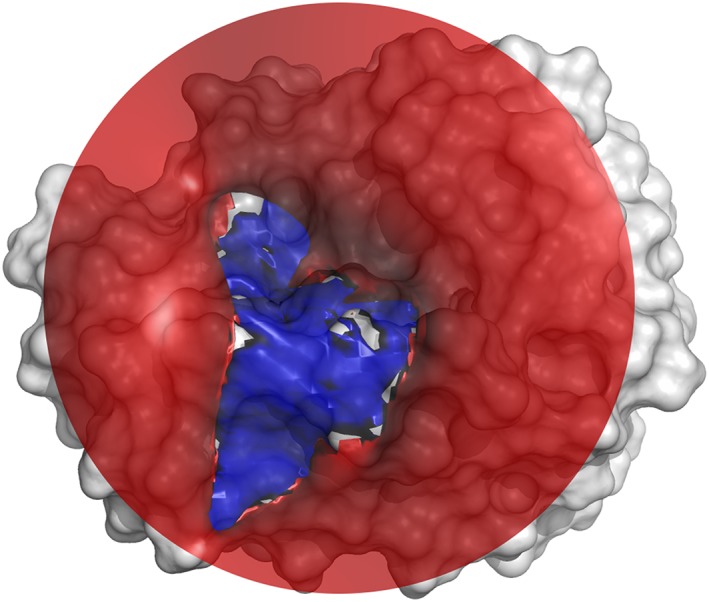
The binding interface of trypsin depicted with the eMIFs of the positive (blue) and negative (red) probe. An energy cutoff of −0.5 kcal/mol was used for the visualization of far‐reaching electrostatic interactions. The eMIF forms a funnel‐like long‐range interaction profile that presumably guides substrates towards an initial encounter complex

This is in line with the notion that protein‐protein recognition follows a 2‐step mechanism. Firstly, an initial encounter complex forms when enzyme and substrate meet. The association rates for this initial encounter complex are largely governed by electrostatics.^80^ An energy funnel pulls substrate and enzyme together and directs the substrate towards the binding site^81^, ^82^, ^83^, ^84^ (Figure 9). In a second step, conformational changes lead to the formation of a compatible binding interface.^85^, ^86^ Here, shape complementarity and flexibility are crucial to enable weak van der Waals interactions and to avoid clashes.

Electrostatics and shape complementarity in context of substrate recognition can be considered rather orthogonal properties resulting in different aspects of substrate recognition.^87^ Thus, we believe that these 2 aspects of substrate recognition can be separated very efficiently by our knowledge‐based approach to analyze substrate readout data as presented in this study. Electrostatic substrate preferences can be characterized very well by binning substrate residues according to their charge. On the other hand, we expect that shape complementarity can be characterized by analyzing substrate recognition within the 3 bins, especially within the neutral bin comprising 15 different neutral amino acid residues. If we can describe the contributions of electrostatics and shape complementarity in a solely physics‐based way, it will be possible to predict the localized specificity and promiscuity of proteases and most likely also of other biomolecular interfaces.

## 5 CONCLUSIONS

A knowledge‐based approach to characterize differences in electrostatic substrate preferences is introduced and applied on 9 homologous serine proteases of the chymotrypsin family. The approach bins known substrate residues into positively charged, negatively charged, and neutral amino acids. Thus, electrostatic preferences in substrate recognition are quantified within subpockets of the binding cleft of the 9 serine proteases and can be compared between different proteases. Similarities and differences in electrostatic preferences can easily be identified on a localized subpocket level but also globally for the complete binding cleft.

On the other hand, eMIFs are calculated in a physics‐based way studying X‐ray structures using the program GRID in combination with user‐defined probes that focus on electrostatics. The binding cleft within the X‐ray structures is delimited by a proximity criterion to known ligands. Calculating the overlap between eMIFs results again in similarities and differences in electrostatic preferences.

Comparing the knowledge‐based and physics‐based similarities and differences in electrostatic preferences, a high correlation between the 2 totally different approaches is found. This implies that the electrostatic part of substrate recognition and substrate specificity can be explained very well by eMIFs.

Due to the long‐range nature of electrostatics, we assume that these electrostatic molecular interactions fields determine the formation of an initial encounter complex between substrates and proteases.

## Supporting information

Figure S1. Workflow for the calculation of the eMIF overlap.
Figure S2. Values calculated with σ = 1 and a grid spacing of 1 Å.
Figure S3. Values calculated with σ = 1 and a grid spacing of 0.5 Å.
Figure S4. Values calculated with σ = 2 and a grid spacing of 1 Å.
Figure S5. Values calculated with σ = 2 and a grid spacing of 0.5 Å.
Figure S*6*: The similarities between the different proteases with histidine as a positive amino acid (upper) and histidine as a neutral amino acid (lower).
Figure S*7*: The positive part of the substrate similarity (upper) and the negative part of the substrate similarity (lower), when considering histidine as an uncharged amino acid.
Figure S*8*: Similarities calculated with webPIPSA and the APBS method for calculating the electrostatic potential. The similarity was calculated at the binding interface, at a point directly at the S1, with a radius of 17 Angstrom, to cover the entire binding site.
Table S1. Correlation of the positive probe for different values of σ and different grid spacing.
Table S2: Correlation of the negative probe for different values of σ and different grid spacing.
Table S 3: The cleavage data of trypsin as supplied in the MEROPS database.
Table S 4: The cleavage data of factor VIIa as supplied in the MEROPS database.
Table S 5: The cleavage data of factor Xa as supplied in the MEROPS database.
Table S 6: The cleavage data of thrombin as supplied in the MEROPS database.
Table S 7: The cleavage data of kallikrein‐1 as supplied in the MEROPS database.
Table S 8: The cleavage data of chymotrypsin as supplied in the MEROPS database.
Table S 9: The cleavage data of elastase‐1 as supplied in the MEROPS database.
Table S 10: The cleavage data of granzyme M as supplied in the MEROPS database.
Table S 11: The cleavage data of granzyme B as supplied in the MEROPS database.
