# Supplementary material for: Electrostatic recognition in substrate binding to serine proteases
Source: J Mol Recognit. 2018 May 22;31(10):e2727. doi: 10.1002/jmr.2727 (PMC6175425; doi:10.1002/jmr.2727)
Supplement: Supplementary file 1 — Figure S1. Workflow for the calculation of the eMIF overlap. Figure S2. Values calculated with σ = 1 and a grid spacing of 1 Å. Figure S3. Values calculated with σ = 1 and a grid spacing of 0.5 Å. Figure S4. Values calculated with σ = 2 and a grid spacing of 1 Å. Figure S5. Values calculated with σ = 2 and a grid spacing of 0.5 Å. Figure S6: The similarities between the different proteases with histidine as a positive amino acid (upper) and histidine as a neutral amino acid (lower). Figure S7: The positive part of the substrate similarity (upper) and the negative part of the substrate similarity (lower), when considering histidine as an uncharged amino acid. Figure S8: Similarities calculated with webPIPSA and the APBS method for calculating the electrostatic potential. The similarity was calculated at the binding interface, at a point directly at the S1, with a radius of 17 Angstrom, to cover the entire binding site. Table S1. Correlation of the positive probe for different values of σ and different grid spacing. Table S2: Correlation of the negative probe for different values of σ and different grid spacing. Table S 3: The cleavage data of trypsin as supplied in the MEROPS database. Table S 4: The cleavage data of factor VIIa as supplied in the MEROPS database. Table S 5: The cleavage data of factor Xa as supplied in the MEROPS database. Table S 6: The cleavage data of thrombin as supplied in the MEROPS database. Table S 7: The cleavage data of kallikrein‐1 as supplied in the MEROPS database. Table S 8: The cleavage data of chymotrypsin as supplied in the MEROPS database. Table S 9: The cleavage data of elastase‐1 as supplied in the MEROPS database. Table S 10: The cleavage data of granzyme M as supplied in the MEROPS database. Table S 11: The cleavage data of granzyme B as supplied in the MEROPS database. [file JMR-31-na-s001.docx]

*Supporting Information for*

*Electrostatic Recognition in Substrate Binding to Serine Proteases*

Birgit J. Waldner,^#,†^ Johannes Kraml,^#,†^ Ursula Kahler,^†^ Alexander Spinn,^†^ Michael Schauperl,^†^ Maren Podewitz,^†^ Julian E. Fuchs,^+,†^ Gabriele Cruciani,^$^ Klaus R. Liedl^*,†^

^†^ Institute of General, Inorganic and Theoretical Chemistry, and Center for Molecular Biosciences Innsbruck (CMBI), University of Innsbruck, Innrain 80-82, 6020 Innsbruck, Tyrol, Austria

^$^ Laboratory of Chemometrics, Department of Chemistry, University of Perugia, Via Elce di Sotto 10, I-60123 Perugia, Italy

*Klaus.Liedl@uibk.ac.at

^#^These authors contributed equally.

## **Calculation of the Overlap**


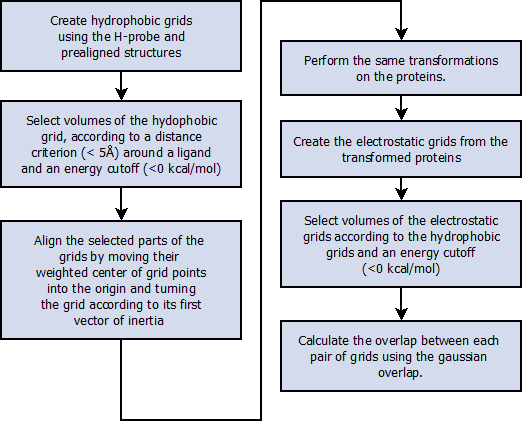


Figure S1. Workflow for the calculation of the eMIF overlap.

## **Results for different sigma values and grid spacing**


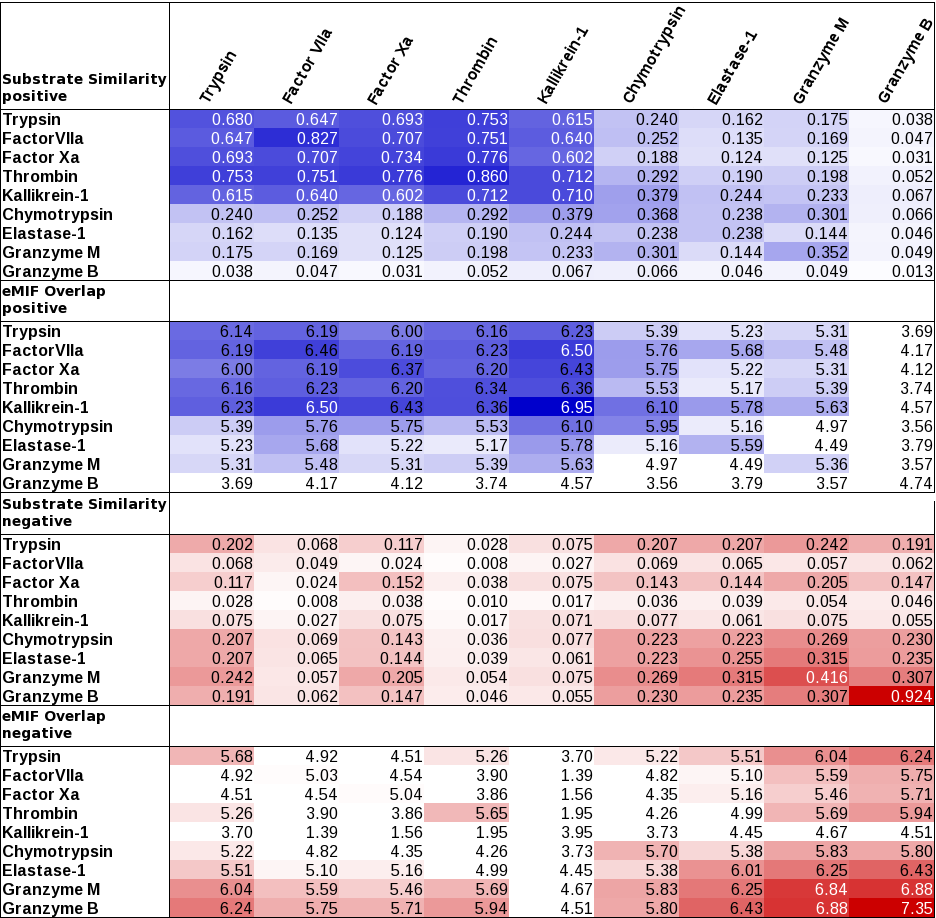


Figure S2. Values calculated with σ=1 and a grid spacing of 1 Å.


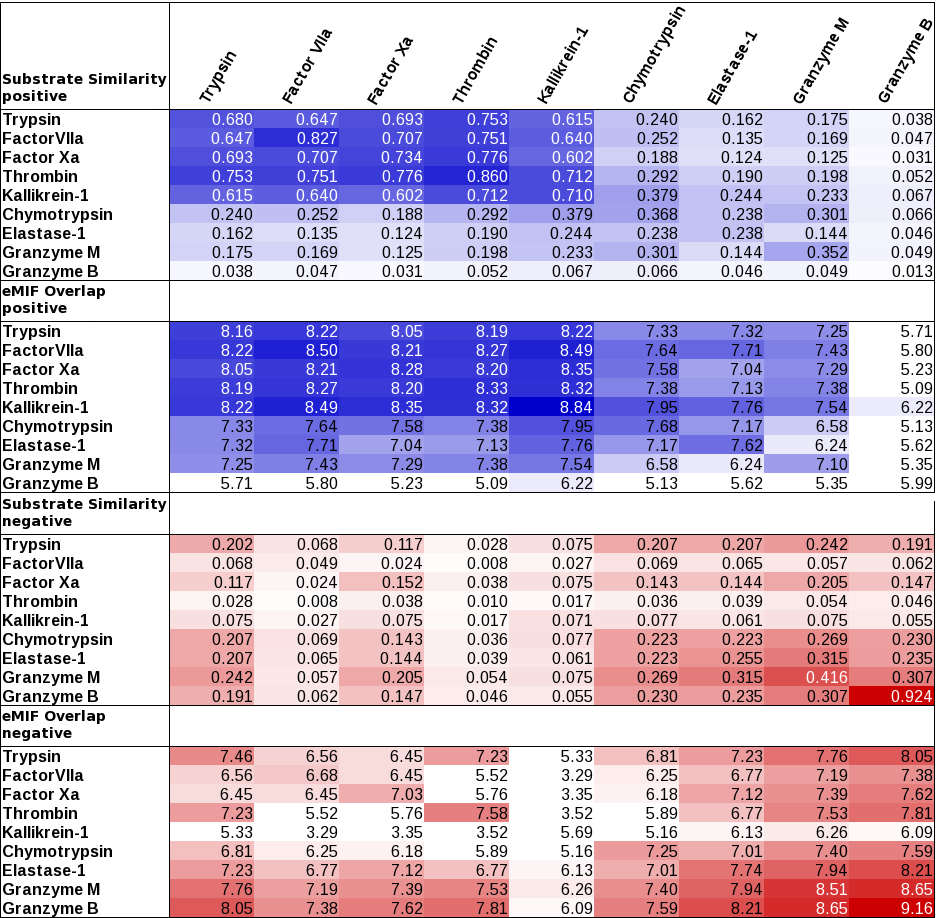


Figure S3. Values calculated with σ=1 and a grid spacing of 0.5 Å.


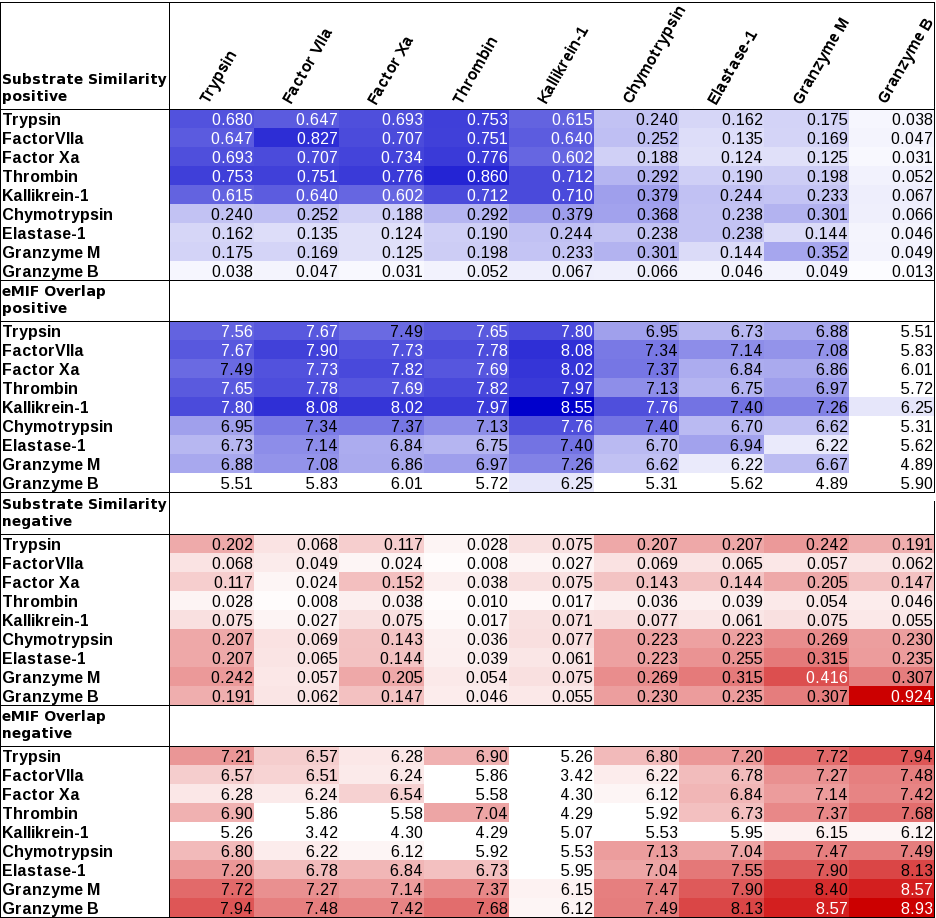


Figure S4. Values calculated with σ=2 and a grid spacing of 1 Å.


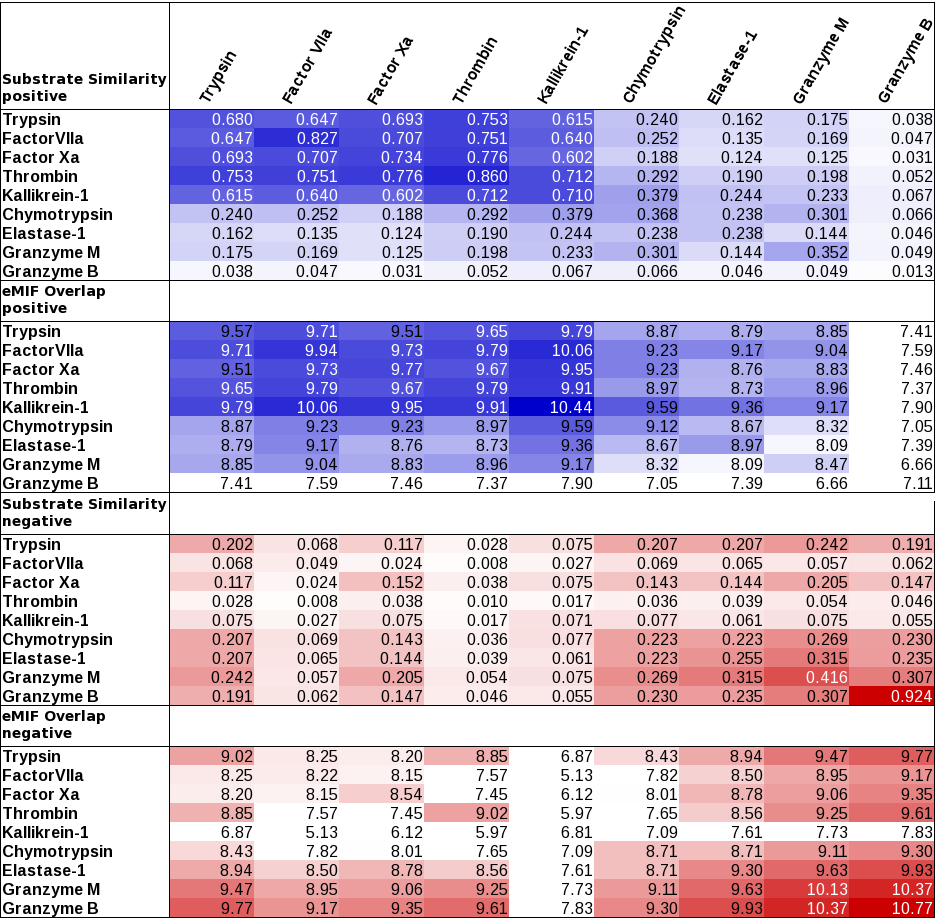


Figure S5. Values calculated with σ=2 and a grid spacing of 0.5 Å.

## **Results for Histidine considered a neutral amino acid**


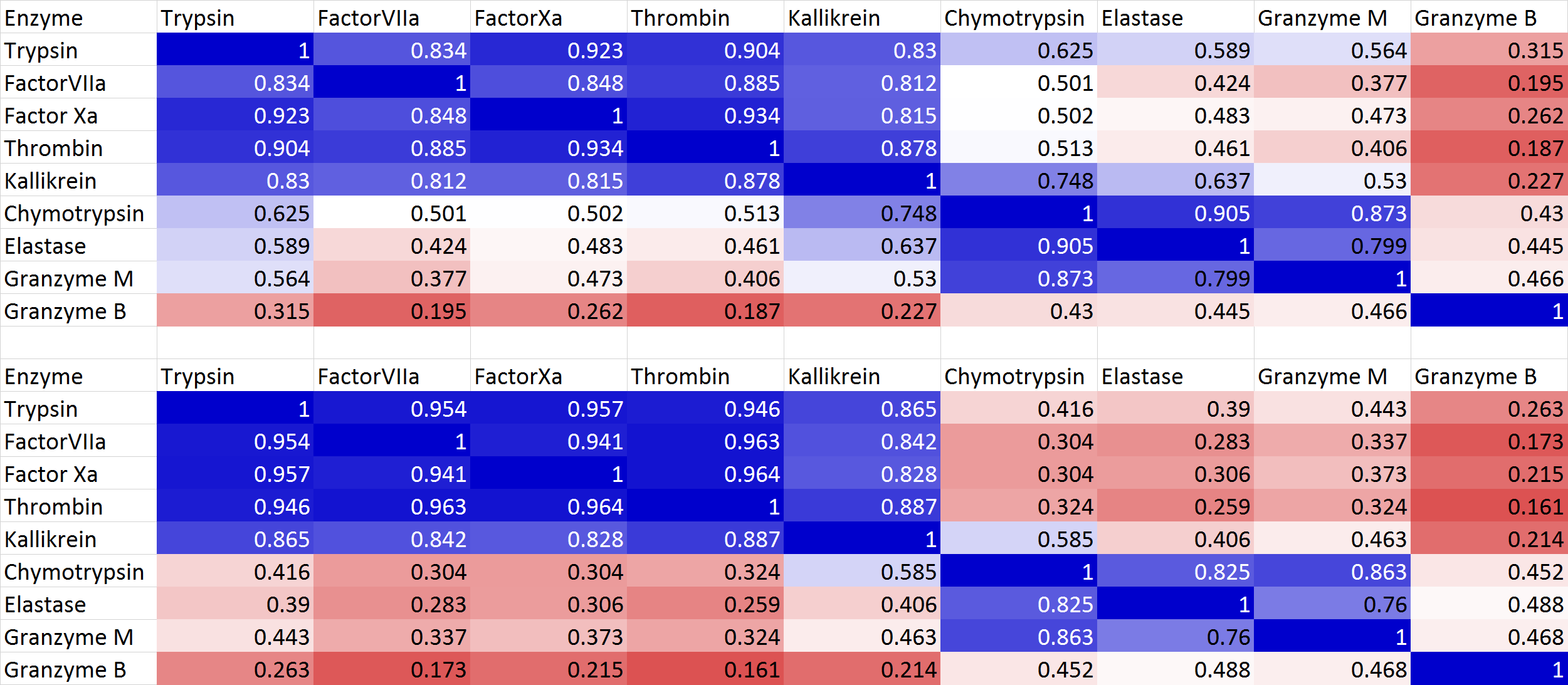


Figure S6: The similarities between the different proteases with histidine as a positive amino acid (upper) and histidine as a neutral amino acid (lower).


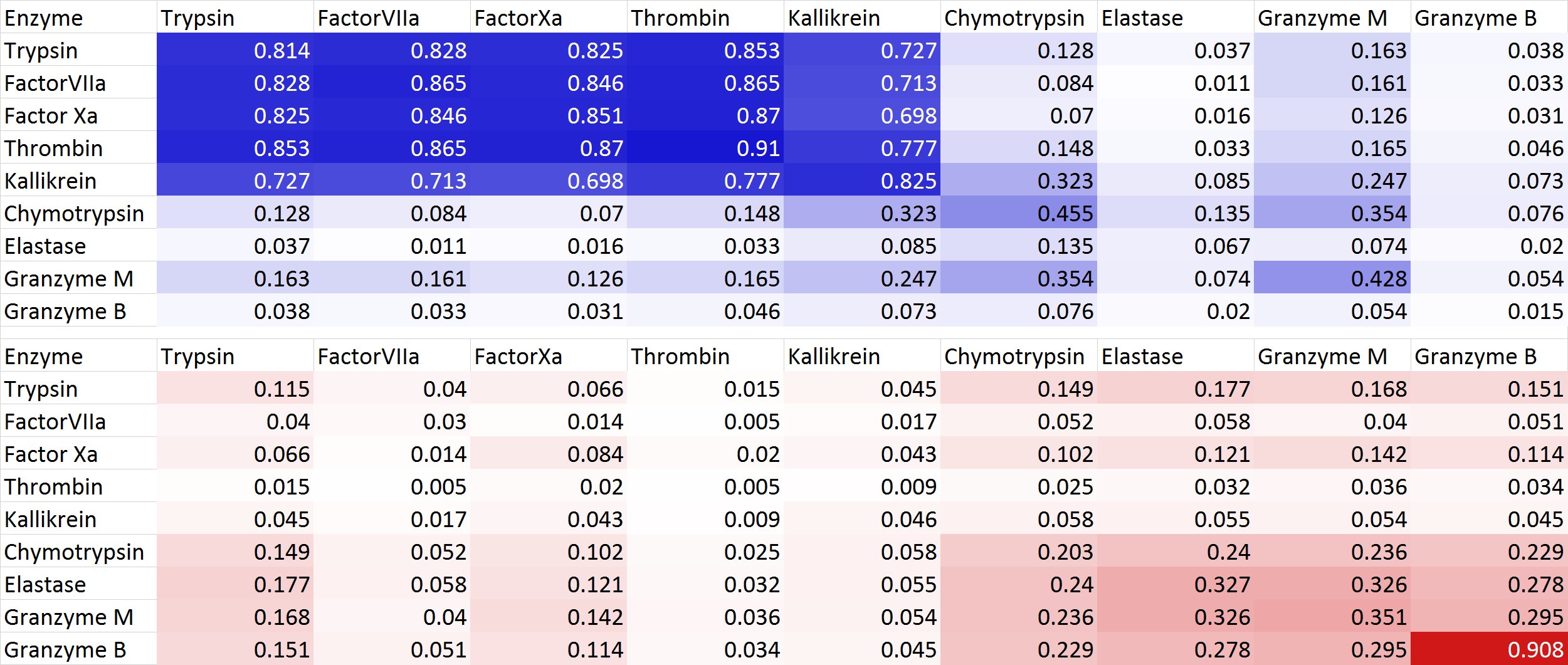


Figure S7: The positive part of the substrate similarity (upper) and the negative part of the substrate similarity (lower), when considering histidine as an uncharged amino acid.


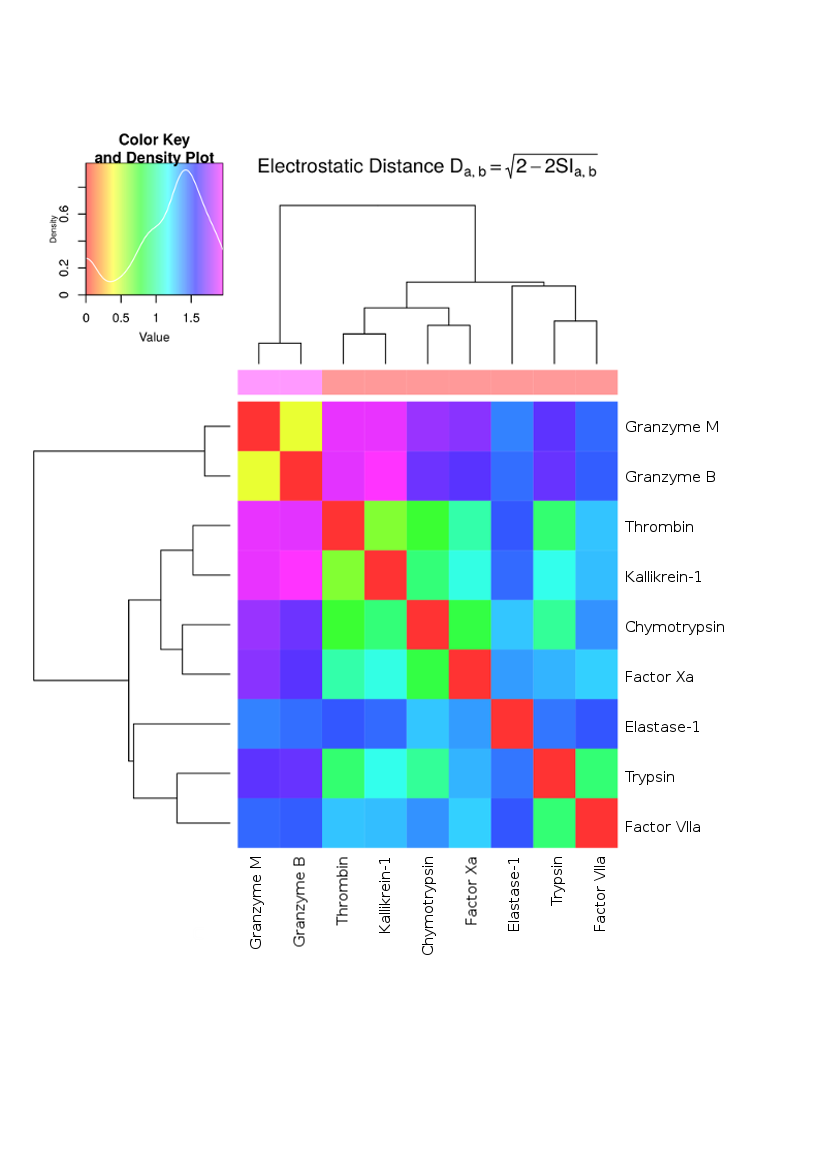


Figure S8: Similarities calculated with webPIPSA and the APBS method for calculating the electrostatic potential. The similarity was calculated at the binding interface, at a point directly at the S1, with a radius of 17 Angstrom, to cover the entire binding site.

## **Correlations**

Table S1. Correlation of the positive probe for different values of σ and different grid spacing.

| Sigma | Grid  Spacing | 1 | 0.5 |
| --- | --- | --- | --- |
| 1 | | 0.87 | 0.90 |
| 2 | | 0.82 | 0.85 |

Table S2: Correlation of the negative probe for different values of σ and different grid spacing.

| Sigma | Grid  Spacing | 1 | 0.5 |
| --- | --- | --- | --- |
| 1 | | 0.58 | 0.56 |
| 2 | | 0.57 | 0.54 |

Correlation of the positive probe with histidine considered an uncharged amino acid: 0.86

Correlation of the negative probe with histidine considered an uncharged amino acid: 0.62

## **WebLogo Data**

Table S 3: The cleavage data of trypsin as supplied in the MEROPS database.

| Amino acid | P4 | P3 | P2 | P1 | P1' | P2' | P3' | P4' |
| --- | --- | --- | --- | --- | --- | --- | --- | --- |
| Gly | 864 | 985 | 1098 | 0 | 908 | 983 | 976 | 841 |
| Pro | 873 | 667 | 842 | 0 | 14 | 545 | 845 | 791 |
| Ala | 1193 | 1191 | 1113 | 0 | 1301 | 1203 | 1146 | 1051 |
| Val | 845 | 1023 | 879 | 0 | 1111 | 1077 | 961 | 949 |
| Leu | 1327 | 1636 | 1614 | 1 | 1348 | 1756 | 1276 | 1317 |
| Ile | 773 | 857 | 795 | 0 | 946 | 967 | 778 | 830 |
| Met | 277 | 314 | 292 | 0 | 348 | 333 | 240 | 260 |
| Phe | 457 | 602 | 579 | 2 | 500 | 601 | 638 | 500 |
| Tyr | 320 | 417 | 385 | 5 | 432 | 420 | 356 | 293 |
| Trp | 104 | 111 | 115 | 1 | 98 | 124 | 94 | 142 |
| Ser | 840 | 889 | 901 | 0 | 901 | 829 | 909 | 888 |
| Thr | 707 | 749 | 686 | 0 | 938 | 777 | 776 | 820 |
| Cys | 156 | 164 | 180 | 1 | 158 | 186 | 226 | 204 |
| Asn | 629 | 556 | 596 | 0 | 602 | 457 | 481 | 467 |
| Gln | 702 | 575 | 663 | 0 | 586 | 515 | 619 | 688 |
| Asp | 1069 | 775 | 575 | 1 | 851 | 734 | 829 | 930 |
| Glu | 1375 | 1051 | 1183 | 3 | 1189 | 751 | 1243 | 1387 |
| Lys | 554 | 612 | 743 | 8200 | 820 | 748 | 662 | 627 |
| Arg | 375 | 293 | 303 | 5555 | 469 | 502 | 437 | 438 |
| His | 249 | 259 | 199 | 1 | 212 | 211 | 197 | 233 |

Table S 4: The cleavage data of factor VIIa as supplied in the MEROPS database.

| Amino acid | P4 | P3 | P2 | P1 | P1' | P2' | P3' | P4' |
| --- | --- | --- | --- | --- | --- | --- | --- | --- |
| Gly | 0 | 1 | 1 | 0 | 0 | 0 | 4 | 4 |
| Pro | 1 | 0 | 1 | 0 | 0 | 0 | 0 | 2 |
| Ala | 0 | 1 | 0 | 0 | 1 | 0 | 0 | 0 |
| Val | 0 | 1 | 1 | 0 | 1 | 4 | 0 | 1 |
| Leu | 3 | 2 | 0 | 0 | 0 | 1 | 0 | 0 |
| Ile | 0 | 1 | 1 | 0 | 3 | 0 | 0 | 0 |
| Met | 1 | 0 | 0 | 0 | 0 | 1 | 0 | 0 |
| Phe | 0 | 1 | 0 | 0 | 0 | 2 | 0 | 0 |
| Tyr | 0 | 0 | 0 | 0 | 0 | 0 | 0 | 0 |
| Trp | 0 | 0 | 0 | 0 | 0 | 0 | 0 | 0 |
| Ser | 0 | 1 | 0 | 0 | 1 | 0 | 1 | 0 |
| Thr | 0 | 0 | 4 | 0 | 1 | 0 | 1 | 0 |
| Cys | 0 | 0 | 0 | 0 | 0 | 0 | 0 | 0 |
| Asn | 1 | 0 | 0 | 0 | 1 | 0 | 0 | 1 |
| Gln | 1 | 1 | 1 | 0 | 0 | 0 | 0 | 0 |
| Asp | 1 | 0 | 0 | 0 | 0 | 0 | 0 | 1 |
| Glu | 0 | 0 | 0 | 0 | 0 | 1 | 0 | 0 |
| Lys | 1 | 0 | 0 | 0 | 1 | 0 | 0 | 0 |
| Arg | 0 | 0 | 0 | 9 | 0 | 0 | 1 | 0 |
| His | 0 | 0 | 0 | 0 | 0 | 0 | 2 | 0 |

Table S 5: The cleavage data of factor Xa as supplied in the MEROPS database.

| Amino acid | P4 | P3 | P2 | P1 | P1' | P2' | P3' | P4' |
| --- | --- | --- | --- | --- | --- | --- | --- | --- |
| Gly | 1 | 3 | 30 | 0 | 1 | 0 | 7 | 13 |
| Pro | 4 | 0 | 10 | 0 | 0 | 0 | 1 | 1 |
| Ala | 14 | 4 | 1 | 0 | 0 | 3 | 2 | 1 |
| Val | 0 | 2 | 0 | 0 | 2 | 12 | 0 | 0 |
| Leu | 6 | 4 | 2 | 0 | 3 | 1 | 1 | 3 |
| Ile | 12 | 0 | 2 | 0 | 8 | 1 | 0 | 5 |
| Met | 1 | 0 | 0 | 0 | 1 | 2 | 2 | 0 |
| Phe | 6 | 8 | 8 | 0 | 0 | 9 | 4 | 1 |
| Tyr | 1 | 1 | 0 | 0 | 0 | 1 | 1 | 0 |
| Trp | 1 | 0 | 2 | 0 | 0 | 0 | 0 | 0 |
| Ser | 2 | 4 | 0 | 0 | 9 | 6 | 5 | 4 |
| Thr | 1 | 1 | 0 | 0 | 12 | 2 | 5 | 2 |
| Cys | 0 | 0 | 0 | 0 | 2 | 0 | 1 | 0 |
| Asn | 0 | 2 | 0 | 0 | 1 | 1 | 2 | 3 |
| Gln | 1 | 5 | 0 | 0 | 2 | 1 | 1 | 2 |
| Asp | 0 | 6 | 1 | 0 | 0 | 2 | 0 | 3 |
| Glu | 3 | 11 | 3 | 0 | 0 | 1 | 5 | 0 |
| Lys | 1 | 3 | 0 | 8 | 1 | 1 | 2 | 3 |
| Arg | 2 | 1 | 0 | 51 | 0 | 0 | 1 | 2 |
| His | 0 | 0 | 0 | 0 | 1 | 0 | 3 | 0 |

Table S 6: The cleavage data of thrombin as supplied in the MEROPS database.

| Amino acid | P4 | P3 | P2 | P1 | P1' | P2' | P3' | P4' |
| --- | --- | --- | --- | --- | --- | --- | --- | --- |
| Gly | 14 | 14 | 20 | 0 | 25 | 8 | 20 | 16 |
| Pro | 14 | 3 | 75 | 0 | 1 | 8 | 10 | 9 |
| Ala | 33 | 15 | 21 | 1 | 28 | 16 | 9 | 13 |
| Val | 11 | 11 | 16 | 0 | 5 | 16 | 10 | 17 |
| Leu | 15 | 7 | 19 | 1 | 10 | 18 | 20 | 11 |
| Ile | 11 | 15 | 7 | 0 | 7 | 9 | 9 | 11 |
| Met | 10 | 4 | 1 | 0 | 1 | 2 | 1 | 0 |
| Phe | 7 | 5 | 4 | 0 | 3 | 13 | 3 | 5 |
| Tyr | 4 | 4 | 0 | 0 | 1 | 9 | 2 | 3 |
| Trp | 2 | 2 | 0 | 0 | 0 | 0 | 0 | 1 |
| Ser | 9 | 14 | 3 | 0 | 45 | 8 | 16 | 17 |
| Thr | 3 | 15 | 4 | 0 | 11 | 10 | 8 | 14 |
| Cys | 0 | 6 | 0 | 0 | 0 | 4 | 0 | 1 |
| Asn | 3 | 8 | 5 | 0 | 0 | 3 | 5 | 8 |
| Gln | 10 | 9 | 7 | 0 | 2 | 11 | 13 | 6 |
| Asp | 0 | 8 | 0 | 1 | 0 | 0 | 1 | 2 |
| Glu | 1 | 5 | 0 | 0 | 0 | 3 | 2 | 3 |
| Lys | 6 | 11 | 1 | 23 | 2 | 11 | 15 | 13 |
| Arg | 1 | 17 | 0 | 159 | 10 | 8 | 10 | 5 |
| His | 2 | 3 | 0 | 1 | 9 | 3 | 6 | 4 |

Table S 7: The cleavage data of kallikrein-1 as supplied in the MEROPS database.

| Amino acid | P4 | P3 | P2 | P1 | P1' | P2' | P3' | P4' |
| --- | --- | --- | --- | --- | --- | --- | --- | --- |
| Gly | 1 | 1 | 0 | 0 | 0 | 0 | 7 | 2 |
| Pro | 1 | 3 | 1 | 0 | 0 | 0 | 2 | 2 |
| Ala | 4 | 7 | 2 | 0 | 1 | 3 | 0 | 5 |
| Val | 0 | 2 | 0 | 0 | 0 | 0 | 2 | 0 |
| Leu | 3 | 0 | 7 | 0 | 1 | 0 | 1 | 2 |
| Ile | 3 | 0 | 0 | 1 | 1 | 2 | 0 | 1 |
| Met | 0 | 0 | 0 | 1 | 0 | 2 | 0 | 0 |
| Phe | 0 | 1 | 8 | 2 | 0 | 1 | 1 | 1 |
| Tyr | 0 | 0 | 1 | 8 | 1 | 1 | 1 | 1 |
| Trp | 0 | 0 | 1 | 2 | 0 | 0 | 0 | 0 |
| Ser | 4 | 4 | 3 | 0 | 13 | 2 | 2 | 2 |
| Thr | 2 | 3 | 1 | 0 | 0 | 4 | 1 | 2 |
| Cys | 1 | 1 | 0 | 0 | 0 | 0 | 0 | 0 |
| Asn | 1 | 1 | 0 | 0 | 1 | 1 | 0 | 1 |
| Gln | 1 | 0 | 1 | 0 | 0 | 1 | 1 | 2 |
| Asp | 0 | 2 | 0 | 0 | 0 | 0 | 0 | 0 |
| Glu | 4 | 1 | 1 | 0 | 0 | 0 | 0 | 0 |
| Lys | 0 | 1 | 1 | 2 | 1 | 2 | 3 | 0 |
| Arg | 0 | 2 | 1 | 15 | 6 | 6 | 3 | 3 |
| His | 0 | 0 | 1 | 0 | 0 | 0 | 1 | 1 |

Table S 8: The cleavage data of chymotrypsin as supplied in the MEROPS database.

| Amino acid | P4 | P3 | P2 | P1 | P1' | P2' | P3' | P4' |
| --- | --- | --- | --- | --- | --- | --- | --- | --- |
| Gly | 125 | 105 | 110 | 0 | 87 | 68 | 111 | 104 |
| Pro | 69 | 30 | 76 | 1 | 8 | 66 | 74 | 78 |
| Ala | 132 | 125 | 91 | 3 | 91 | 90 | 82 | 77 |
| Val | 98 | 73 | 63 | 1 | 83 | 54 | 47 | 37 |
| Leu | 83 | 34 | 112 | 274 | 113 | 94 | 77 | 88 |
| Ile | 37 | 55 | 68 | 2 | 67 | 38 | 41 | 28 |
| Met | 17 | 23 | 5 | 41 | 20 | 17 | 14 | 12 |
| Phe | 33 | 19 | 13 | 280 | 12 | 36 | 31 | 30 |
| Tyr | 18 | 19 | 36 | 296 | 9 | 43 | 43 | 19 |
| Trp | 5 | 3 | 3 | 63 | 5 | 6 | 7 | 9 |
| Ser | 63 | 67 | 67 | 0 | 106 | 69 | 71 | 53 |
| Thr | 39 | 87 | 74 | 1 | 83 | 55 | 63 | 80 |
| Cys | 10 | 12 | 11 | 2 | 14 | 24 | 6 | 41 |
| Asn | 47 | 56 | 43 | 36 | 35 | 53 | 60 | 51 |
| Gln | 22 | 39 | 31 | 8 | 23 | 62 | 31 | 41 |
| Asp | 36 | 44 | 36 | 1 | 44 | 38 | 44 | 57 |
| Glu | 51 | 86 | 50 | 9 | 42 | 62 | 45 | 60 |
| Lys | 62 | 103 | 120 | 2 | 132 | 88 | 110 | 106 |
| Arg | 65 | 57 | 30 | 1 | 46 | 44 | 44 | 33 |
| His | 18 | 8 | 9 | 36 | 9 | 17 | 22 | 21 |

Table S 9: The cleavage data of elastase-1 as supplied in the MEROPS database.

| Amino acid | P4 | P3 | P2 | P1 | P1' | P2' | P3' | P4' |
| --- | --- | --- | --- | --- | --- | --- | --- | --- |
| Gly | 3 | 3 | 4 | 2 | 2 | 3 | 1 | 0 |
| Pro | 3 | 0 | 10 | 0 | 0 | 1 | 3 | 1 |
| Ala | 10 | 7 | 6 | 15 | 3 | 3 | 2 | 1 |
| Val | 3 | 4 | 3 | 11 | 4 | 2 | 4 | 1 |
| Leu | 5 | 3 | 4 | 6 | 4 | 6 | 3 | 4 |
| Ile | 3 | 0 | 1 | 3 | 0 | 0 | 1 | 0 |
| Met | 1 | 1 | 1 | 0 | 1 | 0 | 1 | 2 |
| Phe | 0 | 1 | 4 | 0 | 3 | 3 | 0 | 0 |
| Tyr | 1 | 2 | 1 | 3 | 2 | 3 | 3 | 1 |
| Trp | 0 | 0 | 0 | 0 | 0 | 0 | 0 | 0 |
| Ser | 2 | 5 | 1 | 4 | 5 | 1 | 4 | 6 |
| Thr | 0 | 3 | 0 | 4 | 2 | 1 | 1 | 3 |
| Cys | 2 | 3 | 2 | 1 | 1 | 2 | 2 | 3 |
| Asn | 2 | 1 | 0 | 0 | 1 | 2 | 0 | 1 |
| Gln | 1 | 1 | 1 | 1 | 1 | 3 | 2 | 0 |
| Asp | 0 | 0 | 0 | 0 | 0 | 0 | 1 | 0 |
| Glu | 2 | 5 | 2 | 0 | 3 | 2 | 4 | 7 |
| Lys | 0 | 0 | 2 | 0 | 1 | 0 | 1 | 0 |
| Arg | 0 | 1 | 2 | 0 | 0 | 1 | 0 | 2 |
| His | 0 | 3 | 2 | 1 | 1 | 0 | 1 | 1 |

Table S 10: The cleavage data of granzyme M as supplied in the MEROPS database.

| Amino acid | P4 | P3 | P2 | P1 | P1' | P2' | P3' | P4' |
| --- | --- | --- | --- | --- | --- | --- | --- | --- |
| Gly | 35 | 38 | 152 | 13 | 49 | 106 | 91 | 108 |
| Pro | 113 | 11 | 309 | 6 | 2 | 5 | 97 | 78 |
| Ala | 127 | 145 | 227 | 62 | 212 | 296 | 146 | 159 |
| Val | 60 | 114 | 75 | 7 | 65 | 91 | 77 | 66 |
| Leu | 119 | 86 | 92 | 741 | 45 | 134 | 70 | 65 |
| Ile | 53 | 40 | 22 | 17 | 51 | 46 | 40 | 49 |
| Met | 52 | 33 | 33 | 180 | 33 | 31 | 24 | 22 |
| Phe | 18 | 34 | 17 | 25 | 15 | 79 | 17 | 20 |
| Tyr | 14 | 40 | 25 | 6 | 20 | 79 | 25 | 11 |
| Trp | 6 | 3 | 2 | 0 | 1 | 0 | 4 | 0 |
| Ser | 37 | 118 | 68 | 45 | 244 | 148 | 130 | 135 |
| Thr | 27 | 68 | 48 | 54 | 89 | 57 | 56 | 62 |
| Cys | 9 | 17 | 10 | 32 | 42 | 7 | 11 | 9 |
| Asn | 14 | 54 | 15 | 42 | 79 | 43 | 30 | 50 |
| Gln | 123 | 106 | 52 | 44 | 59 | 34 | 69 | 63 |
| Asp | 6 | 72 | 17 | 3 | 49 | 36 | 137 | 113 |
| Glu | 25 | 251 | 85 | 12 | 63 | 36 | 175 | 158 |
| Lys | 412 | 69 | 84 | 41 | 207 | 92 | 127 | 126 |
| Arg | 72 | 49 | 23 | 28 | 13 | 10 | 12 | 23 |
| His | 39 | 15 | 7 | 4 | 10 | 18 | 10 | 31 |

Table S 11: The cleavage data of granzyme B as supplied in the MEROPS database.

| Amino acid | P4 | P3 | P2 | P1 | P1' | P2' | P3' | P4' |
| --- | --- | --- | --- | --- | --- | --- | --- | --- |
| Gly | 38 | 225 | 204 | 6 | 100 | 208 | 75 | 100 |
| Pro | 136 | 8 | 277 | 2 | 5 | 11 | 64 | 58 |
| Ala | 111 | 158 | 249 | 11 | 291 | 188 | 122 | 140 |
| Val | 459 | 83 | 150 | 3 | 124 | 68 | 200 | 156 |
| Leu | 252 | 108 | 110 | 20 | 113 | 298 | 152 | 147 |
| Ile | 345 | 41 | 25 | 1 | 42 | 85 | 128 | 98 |
| Met | 99 | 50 | 31 | 19 | 39 | 55 | 38 | 53 |
| Phe | 72 | 24 | 49 | 5 | 64 | 77 | 53 | 51 |
| Tyr | 27 | 13 | 14 | 4 | 48 | 74 | 63 | 26 |
| Trp | 9 | 4 | 6 | 6 | 3 | 8 | 7 | 8 |
| Ser | 46 | 213 | 148 | 23 | 374 | 159 | 154 | 124 |
| Thr | 56 | 86 | 110 | 7 | 150 | 68 | 106 | 94 |
| Cys | 13 | 36 | 16 | 5 | 21 | 13 | 12 | 9 |
| Asn | 29 | 66 | 71 | 49 | 71 | 31 | 53 | 54 |
| Gln | 29 | 88 | 132 | 10 | 69 | 66 | 54 | 73 |
| Asp | 26 | 183 | 48 | 1398 | 94 | 165 | 133 | 230 |
| Glu | 67 | 349 | 176 | 261 | 166 | 211 | 281 | 342 |
| Lys | 36 | 60 | 38 | 19 | 62 | 57 | 149 | 78 |
| Arg | 7 | 36 | 9 | 22 | 19 | 17 | 14 | 13 |
| His | 10 | 41 | 10 | 2 | 16 | 12 | 10 | 14 |
